# Collaborative Care to Improve Quality of Life for Anxiety and Depression in Posttraumatic Epilepsy (CoCarePTE): Protocol for a Randomized Hybrid Effectiveness-Implementation Trial

**DOI:** 10.2196/59329

**Published:** 2024-11-13

**Authors:** Heidi M Munger Clary, Beverly M Snively, Christian Cagle, Richard Kennerly, James N Kimball, Halley B Alexander, Gretchen A Brenes, Justin B Moore, Robin A Hurley

**Affiliations:** 1 Department of Neurology Wake Forest University School of Medicine Winston-Salem, NC United States; 2 Research and Academic Affairs W. G. Heffner Veterans Affairs Medical Center Salisbury, NC United States; 3 Department of Biostatistics and Data Science Wake Forest University School of Medicine Winston-Salem, NC United States; 4 Mental Health and Behavioral Sciences W. G. Heffner Veterans Affairs Medical Center Salisbury, NC United States; 5 Department of Psychiatry Wake Forest University School of Medicine Winston-Salem, NC United States; 6 Department of Internal Medicine Section of Gerontology and Geriatric Medicine Wake Forest University School of Medicine Winston-Salem, NC United States; 7 Department of Implementation Science Wake Forest University School of Medicine Winston-Salem, NC United States; 8 Department of Radiology Wake Forest University School of Medicine Winston-Salem, NC United States

**Keywords:** integrated care, mental health, seizures, psychiatric comorbidity, neurology clinic, epilepsy

## Abstract

**Background:**

Anxiety and depression in people with epilepsy are common and associated with poor outcomes; yet, they often go untreated due to poor mental health specialist access. Collaborative care is an integrated care model with a strong evidence base in primary care and medical settings, but it has not been evaluated in neurology clinics. Evaluating implementation outcomes when translating evidence-based interventions to new clinical settings to inform future scaling and incorporation into real-world practice is important.

**Objective:**

The Collaborative Care for Posttraumatic Epilepsy (CoCarePTE) trial aims to evaluate the effectiveness (improvement in emotional quality of life) and implementation of a collaborative care intervention for people with anxiety or depressive symptoms and posttraumatic epilepsy.

**Methods:**

CoCarePTE is a 2-site, randomized, single-blind, hybrid type 1 effectiveness-implementation trial that will randomize 60 adults to receive either neurology-based collaborative care or usual care. Adults receiving neurological care at participating centers with anxiety or depressive symptoms and a history of at least mild traumatic brain injury before epilepsy onset will be enrolled. The collaborative care intervention is a 24-week stepped-care model with video or telephone calls every 2 weeks by a care manager for measurement-based anxiety and depression care, seizure care monitoring, and brief therapy intervention delivery. This is supplemented by antidepressant prescribing recommendations by psychiatrists for neurologists via case conferences and care manager–facilitated team communication. In step 2 of the intervention, individuals with <50% symptom reduction by 10 weeks will receive an added 8-session remote cognitive behavioral therapy program. The study is powered to detect a moderate improvement in emotional quality of life. As a hybrid type 1 trial, effectiveness is the primary focus, with the primary outcome being a change in emotional quality of life at 6 months in the intervention group compared to control. Secondary effectiveness outcomes are 6-month changes in depression, anxiety, and overall quality of life. Implementation outcomes, including fidelity, acceptability, feasibility, and appropriateness, are evaluated before implementation and at 3 months. The primary effectiveness analysis will compare changes in emotional quality of life scores from baseline to 6 months between the intervention and control arms using multiple linear regression modeling, adjusting for study site and using an intent-to-treat approach.

**Results:**

Enrollment commenced in 2023, with modifications in the inclusion and exclusion made after the first 6 enrollees due to slow recruitment. Enrollment is expected to continue at least into early 2025.

**Conclusions:**

The CoCarePTE trial is novel in its use of a hybrid effectiveness-implementation design to evaluate an evidence-based mental health intervention in epilepsy, and by incorporating seizure care into a collaborative care model. If a significant improvement in emotional quality of life is found in the intervention group compared to usual care, this would support next step scaling or clinical implementation.

**Trial Registration:**

ClinicalTrials.gov NCT05353452; https://www.clinicaltrials.gov/study/NCT05353452

**International Registered Report Identifier (IRRID):**

DERR1-10.2196/59329

## Introduction

### Background

Anxiety and depression in epilepsy are highly prevalent, and they are stronger independent predictors of poor quality of life than seizure frequency [1]. They are particularly relevant to people with posttraumatic epilepsy (PTE), including veterans. Anxiety and depression are significantly more common among people with epilepsy than the general population, with pooled point prevalence of 20.2% for anxiety and 22.9% for depression in a recent meta-analysis [2] and lifetime prevalence of 30% to 35% demonstrated in a population-based study [3]. In addition to being associated with poor quality of life, anxiety and depression in epilepsy are associated with increased mortality from suicide, higher health care costs, cognitive dysfunction, adverse effects from medication, and poor seizure outcomes [1,4-8].

PTE (epilepsy caused by traumatic brain injury [TBI]) is more common in prevalent versus incident epilepsy samples, indicating a tendency for greater chronicity or severity than many other causes of epilepsy, and it is common, accounting for up to 20% of prevalent epilepsies [9]. PTE is a significant predictor of anxiety and depression 2 years after moderate to severe TBI [10]. Despite the impact of anxiety and depression in epilepsy on quality of life and other outcomes, including suicide risk, these comorbidities are underrecognized and undertreated [11-13]. Neurologists face significant barriers to arranging specialty mental health access for people with epilepsy; however, most are willing to prescribe antidepressants [14,15]. Prior work demonstrates that usual neurology care for anxiety and depression in epilepsy does not improve quality of life [16], and symptoms may persist despite a prescribed antidepressant [17]. However, people with epilepsy desire treatment for anxiety and depression in the neurology setting and are willing to participate in research [18,19]. Thus, to improve quality of life among individuals with PTE and anxiety or depression, enhanced care interventions should be studied in the neurology setting.

Collaborative care models for managing anxiety and depression are highly effective in nonpsychiatric settings and improve quality of life [20]. These models were successfully implemented in US Department of Veterans Affairs (VA) primary care and various subspecialty settings [21-23], and a home-based depression program in epilepsy was beneficial [24]; yet, collaborative care has not been investigated in neurology clinics. As anxiety and depression in epilepsy are particularly relevant to people with PTE and considering the particular risk for anxiety and depression in PTE, it is important to study neurology collaborative care implementation to improve quality of life, anxiety, and depression in PTE.

Effectiveness-implementation hybrid trial designs are optimal for studying interventions (such as collaborative care) that have a strong evidence base in other health conditions but are being translated to a new setting and disease area [25]. This approach can speed the dissemination of evidence-based interventions into new settings by initially testing effectiveness in that setting (the degree of benefit derived in real-world circumstances rather than in the ideal conditions typical of efficacy trials). Moreover, hybrid type 1 effectiveness-implementation designs can further support future dissemination of effective interventions by emphasizing effectiveness outcomes and also collecting information on implementation [25]. This study design is a good fit for evaluating collaborative care in the new setting (neurology clinics) and population (individuals with PTE). Thus, we describe a protocol for a hybrid 1 randomized trial to evaluate collaborative care for PTE (CoCarePTE) to test the hypotheses outlined in the next subsection.

### Hypotheses

The primary hypothesis to be tested in this study is whether collaborative care improves emotional quality of life at 6 months in adults with anxiety or depressive symptoms and PTE compared with usual neurology care (effectiveness). A clinically significant improvement in emotional quality of life (defined as a moderate change—20 units [26]—in the emotional quality of life subscale of the Quality of Life in Epilepsy-31 [QOLIE-31] scale), compared to control, would warrant immediate clinical implementation of the intervention. The secondary hypothesis overall (implementation) is that the fidelity of the intervention, defined as the proportion of those receiving the collaborative care intervention who attend a majority of care management video or telephone calls in 12 weeks, is very good (ie, >60%). This level of fidelity was chosen based on the clinical judgment of the investigators because it was felt that a fidelity of <60% would definitely warrant modification of the intervention as a next step.

### Aims

The aims of the CoCarePTE trial are as follows:

1. To evaluate the effectiveness of a neurology-based, video and telephone delivered, 24-week stepped collaborative care intervention adapted from existing local programs among 60 adults with PTE and anxiety or depressive symptoms (the primary effectiveness outcome is group-level change in emotional quality of life at 6 months; and secondary effectiveness outcomes are group-level changes in depressive symptoms, anxiety symptoms, and overall quality of life at 6 months)
2. To assess early implementation outcomes of the neurology-based collaborative care intervention, with primary implementation outcome being intervention fidelity (patient participant attendance at intervention visits in the first 12 weeks; patient- and provider-level acceptability, feasibility, and appropriateness of the intervention will also be evaluated)
3. To explore potential mediators and moderators of the effectiveness of collaborative care (primary and secondary effectiveness outcomes), including seizure factors (seizure frequency and severity) and treatment factors (medication adherence and side effects)

## Methods

### Study Design

This is a single-blind, 2-site civilian and VA randomized type 1 hybrid effectiveness-implementation trial comparing a 24-week neurology-based, stepped collaborative care intervention for anxiety and depression with usual neurology care. The study will include adults with epilepsy, anxiety or depressive symptoms, and history of a mild (or worse) TBI before their first seizure. Individuals will undergo site-stratified randomization at a 1:1 ratio to receive either collaborative care or usual neurology care. Effectiveness outcomes will be collected remotely by blinded outcome assessors.

The study has been registered at ClinicalTrials.gov (NCT05353452).

### Settings

This study is being conducted at Atrium Health, a large academic medical center in the southeastern United States affiliated with the Wake Forest University School of Medicine, and at the WG (Bill) Hefner Salisbury VA Medical Center. Both the civilian and VA sites have multiple neurology or epilepsy clinic locations that serve patients from western and southwestern North Carolina along with surrounding areas. Separate intervention teams and research staff recruit participants and deliver the intervention to randomized enrollees at each main site, but the overall study principal investigator (PI) and blinded outcome assessors serve roles at both sites via dual credentialing. Overall study oversight is based at Atrium Health Wake Forest Baptist Neurology, the major academic hub for Wake Forest University School of Medicine neurology research.

### Ethical Considerations

The study has been approved by the institutional review boards (IRBs) of the Wake Forest University School of Medicine (00084191) and the WG (Bill) Hefner Salisbury VA Medical Center (1679395-14). As the study is funded by the Department of Defense (W81XWH2210630), the study protocol was also reviewed and approved by the Department of Defense Office of Human Research Oversight; and the study protocol was also reviewed and approved by the Department of Defense Office of Human Research Oversight; protocol amendments were approved by both IRBs and communicated to the Office of Human Research Oversight. The trial will be carried out in accordance with the International Council for Harmonisation Good Clinical Practice. Full informed consent is obtained from participants before enrollment and baseline data collection and includes permission to transmit deidentified data to the Federal Interagency Traumatic Brain Injury Research (FITBIR) repository for data sharing. All research activities are conducted in as private a setting as possible, and study data are securely stored at each clinical site in locked offices or password-protected secure data systems to which only approved study team members have access. Individual participants and their research data are identified by a unique study ID number, with linkage code stored securely in local site files with password-protected access limited to appropriate study team members. REDCap (Research Electronic Data Capture; version 14.0.21; Vanderbilt University), a 21 Code of Federal Regulations Part 11–compliant data capture system, is used for data collection and storage for analysis. Participants receive a US $40 incentive for completing enrollment and randomization and US $20 each for completing 3- and 6-month outcome procedures.

### Participants and Recruitment Methods

#### Inclusion and Exclusion Criteria

Textbox 1 summarizes the study inclusion and exclusion criteria. Adults receiving neurology care at participating centers are included if they meet the study definition of PTE and exhibit sufficient anxiety or depressive symptoms at screening. To meet study criteria for PTE, the following two criteria must be met: (1) diagnosis of epilepsy by the treating neurology provider based on their clinical impression or ictal or interictal electroencephalogram findings (epileptologist investigator review of documented medical record required for verification) and (2) mild or worse TBI before the first seizure. The presence of mild or worse TBI is assessed based on the Mid-Atlantic Mental Illness Research, Education, and Clinical Center assessment of TBI screener [27], and the temporal relationship of the earliest TBI with seizure onset is evaluated using 3 questions from the National Institute of Neurological Disorders and Stroke (NINDS) PTE screening form. The Mid-Atlantic Mental Illness Research, Education, and Clinical Center assessment of TBI screener is a 5-question validated screener to detect a history of mild or worse TBI [27]. The presence of anxiety or depressive symptoms is determined by scores on the Generalized Anxiety Disorder-7 (GAD-7) and Neurological Disorders Depression Inventory for Epilepsy (NDDI-E) instruments, which are well-validated anxiety and depression screeners in epilepsy. Initial cutoffs for inclusion were GAD-7 score ≥10 or NDDI-E score >15, based on original validation literature [28,29], but these were modified to GAD-7 score ≥8 and NDDI-E score >13 after the first 6 enrollments to facilitate recruitment and to align with more recent meta-analyses of these instruments in epilepsy suggesting refined optimal cutoffs [30-32]. Individuals are excluded if they are not good candidates for collaborative care based on having a prior suicide attempt or active suicidal ideation, active psychiatry care without the ability to benefit from the collaborative care intervention, inadequate cognition to complete self-report instruments as part of measurement-based care and research participation, unstable medical problems, or unstable substance misuse. Individuals currently participating in an intervention research study are also excluded.

**Participant inclusion and exclusion criteria.**

**Inclusion criteria**

- Diagnosis of posttraumatic epilepsy based on neurology clinician impression or electroencephalogram-based diagnosis and at least mild traumatic brain injury before seizure onset
- Anxiety or depressive symptoms (initial inclusion cutoffs of Generalized Anxiety Disorder-7 [GAD-7] score ≥10 or Neurological Disorders Depression Inventory for Epilepsy [NDDI-E] score ≥16 were active during enrollment of the first 6 participants but were reduced to GAD-7 score ≥8 or NDDI-E score ≥14 thereafter)
- Aged ≥18 y
- Willing and interested in participating
- Neurology care at a study site

**Exclusion criteria**

- Inadequate cognition to complete self-report instruments
- Active suicidal ideation
- Past suicide attempt
- Unstable drug or alcohol misuse
- Unstable or progressive medical condition
- Current participant in an intervention study
- Active ongoing psychiatry treatment *without potential to benefit from collaborative care intervention* (the additional component of this exclusion, presented in italics, was added after enrollment of the first 6 participants; this was determined based on investigator judgment as to whether the pre-enrollment psychiatry care was less intensive than the potential study intervention; individuals receiving less intensive psychiatry care [eg, stable medication or medication with no case management or therapy intervention] were considered eligible)

#### Recruitment Methods and Informed Consent Process

Multiple recruitment methods are used at both sites, including neurology clinician referrals; prescreening of scheduled neurology clinic visits with previsit communication to neurologists or follow-up letters and telephone calls; flyers, brochures, cards, and posters advertising the study in neurology clinics; engagement with epilepsy nursing and patient advocacy stakeholders to refer potential participants; electronic recruitment messages in the patient portal (civilian site only); and education and information about the study disseminated to neurology providers via virtual meetings, email, and other communications. Other tools for clinicians available at the civilian site include a patient visit instructions text tool to enable patient self-referral and electronic screening for eligibility, clinician letters mailed to potentially eligible patients identified via data warehouse data pull, and study screening consent and initial screening in the electronic health record (EHR) and embedded in standard care procedures (GAD-7 and NDDI-E screeners attached to all visits at the adult epilepsy clinic) using methods similar to the research team’s prior work [33]. At the VA site, prior participants in the Veterans Integrated Service Network 6 Mid-Atlantic Mental Illness Research, Education, and Clinical Center Post-Deployment Mental Health Study [34] who agreed to be contacted for future studies are also screened for potential eligibility and contacted if potentially eligible.

Individuals referred for screening or who express interest will first complete a very brief electronic or verbal screening consent followed by electronic or verbal study screening questions to determine eligibility. Electronic screening questions may be initiated in the EHR for individuals who expressed interest via EHR screening consent or in REDCap [35]. Those individuals found to be fully eligible and interested then complete a full informed consent process, either on paper at the start of an in-person enrollment visit (primarily at the VA) or via local ethics board–approved electronic (DocuSign) or mailed remote informed consent procedures.

### Research Procedures and Randomization

#### Overview

**Figure 1:**
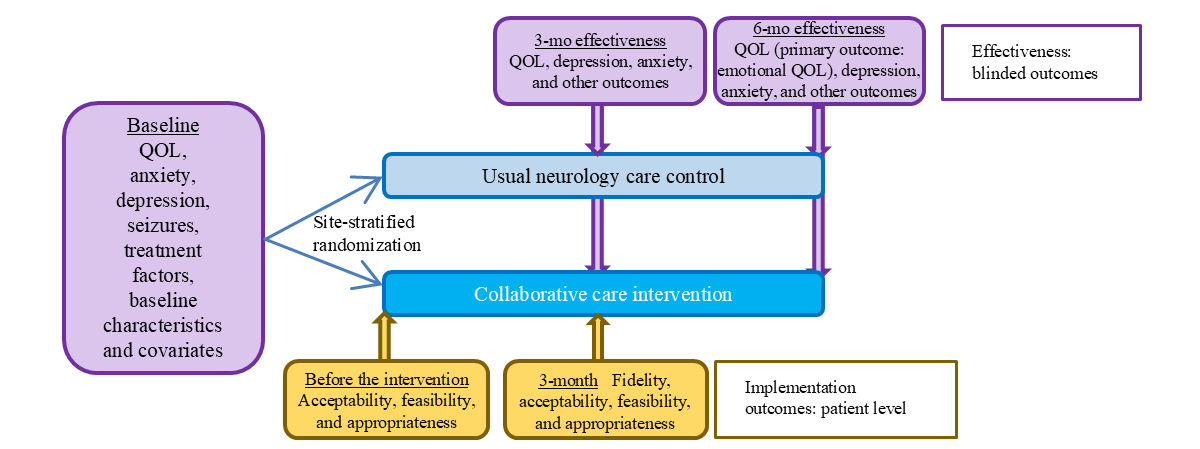
Study design and outcome assessment for patient-level outcomes. QOL: quality of life.

Figure 1 shows an overview of the study procedures. After full informed consent, baseline data collection procedures are conducted by the main site study coordinator either in person or remotely (via telephone and, in some cases, via electronic survey for the self-report instruments). Immediately after baseline data collection is completed, participants are randomized by the study coordinator (via a randomization button in REDCap [35], with the site-stratified blocked randomization scheme having been uploaded by the study statistician in REDCap and concealed from all other study team members until the moment of randomization). Variable block sizes are used, with the randomization sequence generated using R (R Foundation for Statistical Computing) [36]. Participants randomized to the collaborative care intervention group then complete the preintervention implementation outcomes. All participants undergo remote 3- and 6-month effectiveness outcome assessment by a blinded outcome assessor, and this blinded outcome assessment is followed by a supplementary unblinded outcome collection by the main site study coordinator. Blinded assessors have restricted access to the study database and other electronic files to prevent unblinding, and scripts are used by the unblinded staff at outcome reminder calls and by the blinded assessors at the start of outcome assessments to remind participants to refrain from mentioning study group allocation. Data collected by the unblinded coordinator during unblinded assessments include implementation outcomes, medications, and information about any changes in mental health treatment. During the study design and planning process, efforts were made to select study measures aligned with NINDS epilepsy or TBI common data elements.

#### Baseline Data Collection Instruments

At baseline, all effectiveness measures and potential mediators (Table 1) not already collected during screening are administered. The following additional baseline information is also collected: detailed demographics; epilepsy history, including epilepsy type, prior antiseizure medications, cause or risk factors, and prior epilepsy surgeries (aligned with the NINDS epilepsy common data elements). TBI history is assessed via the Ohio State University TBI Short Form [37], while additional core common data elements are evaluated from the FITBIR injury history form and general medical history. The Ohio State University TBI Short Form is a reliable and valid brief structured interview evaluating a history of exposure to TBI, including 5 initial screening questions, and gathering additional information on loss of consciousness, memory gap, and age at injury for each specific incident and any repeated injuries [37]. In addition to the injury history, general medical history and demographics are collected using components of the respective FITBIR forms, including the TBI core common data elements [38]. Epilepsy type (focal, generalized, or unknown) and seizure types are aligned with the current International League Against Epilepsy seizure type and epilepsy classification and the American Academy of Neurology quality measure for seizure type [39-41]. Substance use is evaluated using the Alcohol, Smoking, and Substance Involvement Screening Test, a reliable and valid measure that has 11 screening items for any use across different substance categories, followed by up to 6 additional questions to assess for problematic use among those categories having a positive screen [42]. For each substance area, scores are rated as lower, moderate, or high risk on a scale ranging from 0 to 39, with scores of <4 categorized as lower risk, 4 to 26 as moderate risk, and >26 as high risk. Cognition is evaluated using 5 items from the Brief Test of Adult Cognition by Telephone, a validated cognitive instrument and highly recommended NINDS TBI common data element designed specifically for remote administration via telephone [43]. These items take 5 to 15 minutes to administer.

**Table 1:** Effectiveness end points and exploratory potential mediators (seizure and treatment factors).

Concept		Measure	Administration or collection method	Number of items	Score range^a^ (if applicable)	Notes about administration
**Effectiveness (aim 1)**						
	Emotional quality of life: primary effectiveness outcome	QOLIE-31^b^ (emotional quality of life subscale) [44]	Self-report	5	0-100	—^c^
	Epilepsy quality of life^d^	QOLIE-31 [44]	Self-report	31	0-100	—
	Depressive symptoms^d^	Beck Depression Inventory–Second Edition [45]	Self-report	21	0-63	—
	Anxiety symptoms^d^	Beck Anxiety Inventory [46]	Self-report	21	0-63	—
	Depression and anxiety diagnoses	Mini International Neuropsychiatric Interview for Diagnostic and Statistical Manual of Mental Disorders, Fifth Edition^e^ [47]	Structured diagnostic interview	Modules A, C-H, K, N, and O	—	—
	Major depressive episode screener	Neurological Disorders Depression Inventory for Epilepsy^f^ [29]	Self-report	6	6-24	—
	Generic quality of life	SF-36^g^ [48]	Self-report	36	0-100	10 items are identical to QOLIE-31 items; these are administered with QOLIE-31, and the responses are used for SF-36 scoring along with the other 26 SF-36 items
**Potential mediators (exploratory aim 3)**						
	Seizure frequency	Seizure diary: NINDS^h^ common data elements diary [49]; seizure frequency: American Academy of Neurology epilepsy quality measures seizure frequency categories [41]	Participant paper diary entries reported to study coordinator via interview; interview	—; 2 per seizure type	—	Date, number, and seizure type for each seizure during the time frame; seizure frequency and time since last seizure categories for each seizure type
	Seizure severity	Liverpool Seizure Severity Scale [50]	Self-report	1-13	0-100	—
	Adverse effects	Liverpool Adverse Events Profile [51]	Self-report	19-21	19-48	—
	Medication adherence	Pharmacy records	Record review	—	—	Number of actual refills and number of refills expected for 6 months before enrollment and in each 3-month follow-up time frame

^a^Higher scores indicate higher quality of life for the Quality of Life in Epilepsy-31 (QOLIE-31) emotional quality of life subscale, total QOLIE-31, and Short Form Health Survey-36. Higher scores on the other scaled instruments indicate worse depression, anxiety, seizure severity, or adverse effects.

^b^QOLIE-31: Quality of Life in Epilepsy-31

^c^Not applicable.

^d^Secondary effectiveness outcomes.

^e^Administered only at baseline and 6 months (other measures administered at baseline and at 3 and 6 months).

^f^Screener for the presence of a current major depressive episode.

^g^SF-36: Short Form Health Survey-36.

^h^NINDS: National Institute of Neurological Disorders and Stroke.

### Measures and End Points

#### Effectiveness: Quality of Life, Anxiety, and Depression

The primary outcome is the emotional quality of life subscale of the QOLIE-31 [44], which is identical to the emotional quality of life subscale of the Short Form Health Survey-36 [48] generic quality of life measure; thus, the outcome is of relevance to communities of people with TBI consisting of both those with epilepsy and those without epilepsy. The specific primary outcome is group-level change in emotional quality of life from baseline to 6 months. Secondary effectiveness outcomes are depressive symptoms measured by the Beck Depression Inventory–Second Edition [45], anxiety symptoms measured by the Beck Anxiety Inventory [46], and overall epilepsy-specific quality of life (QOLIE-31 total score) [44]. Similar to the primary outcome, these secondary outcome measures will be evaluated as group-level changes at 6 months. Table 1 outlines details of the effectiveness outcome measures, including the number of items and administration characteristics.

Exploratory effectiveness outcomes include generic overall quality of life (Short Form Health Survey-36) [48]; *Diagnostic and Statistical Manual of Mental Disorders, Fifth Edition*, anxiety and depression diagnoses measured by the Mini International Neuropsychiatric Interview (MINI) [47] for Diagnostic and Statistical Manual of Mental Disorders, Fifth Edition; and epilepsy-specific depression screening status measured by the NDDI-E [30]. At baseline, we administer the mood disorder MINI modules (A [depression], C [manic episodes related to bipolar disorder], and K [psychosis]) as well as the following anxiety and anxiety-related modules: D (panic disorder), E (agoraphobia), F (social anxiety disorder), G (obsessive-compulsive disorder), H (posttraumatic stress disorder), and N (generalized anxiety disorder), along with module O to rule out organic causes. At 6 months, the mood disorder–related modules as well as the panic, generalized anxiety, and posttraumatic stress disorder modules are readministered along with module O for all participants. The agoraphobia and social anxiety disorder modules are repeated at 6 months if a current diagnosis is present at baseline.

#### Potential Mediators: Seizure Frequency, Seizure Severity, and Treatment Factors

Seizure frequency and severity measures are collected at baseline and at 3 and 6 months to explore as potential mediators of the effect of collaborative care. Seizure frequency is collected via a daily seizure diary (NINDS common data elements seizure diary) provided to participants at enrollment, with diary entries collected by telephone at outcome assessments by the blinded outcome assessor. Seizure frequency and time since last seizure are also classified using the American Academy of Neurology epilepsy quality measures seizure frequency categories at baseline and each outcome assessment [41]. Seizure severity is rated using the Liverpool Seizure Severity Scale [50]. Table 1 contains additional information about the administration characteristics of these seizure- and treatment-related end points.

Medication adherence and side effects are also collected to explore as potential mediators. The Liverpool Adverse Events Profile [51] is used to evaluate medication side effects, and pharmacy records are reviewed to evaluate medication adherence for all standing antiseizure medications and psychotropic medications during the study. Past adherence is assessed at baseline based on a review of prescription fills in the 6 months before enrollment; actual refills will be compared to expected refills in both the pre-enrollment and on-study periods.

#### Other Exploratory Health Outcomes, and Other Measures Collected at Follow-Up

Major health care use events, including hospitalizations and emergency visits, will be collected at 3 and 6 months via participant report and supplemented by EHR review. Mental health treatments received as part of the intervention or outside of the intervention will be collected and tracked by an unblinded study team member. The GAD-7 and Patient Health Questionnaire-9 (PHQ-9) are valid and reliable measures of anxiety and depression severity with 7 and 9 scored items, respectively [28,52]. These instruments are used for symptom monitoring in the intervention group and collected at screening and baseline and at 3- and 6-month outcome assessments for all participants. Scores range from 0 to 21 and 0 to 27 for the GAD-7 and PHQ-9, respectively, with higher scores indicating worse symptoms.

#### Implementation

The implementation data collected emphasize early implementation outcomes, with a focus on fidelity (primary implementation outcome), feasibility, acceptability, and appropriateness (Table 2). The primary fidelity measure is the proportion of intervention group participants who attend at least 50% of the care management calls in the first 12 weeks of intervention delivery. This 50% metric was determined based on the consideration of the investigators because it was felt that individuals attending <50% of the visits would be very unlikely to benefit from the intervention. Rates of overall attendance at collaborative care calls are also collected along with various process measures at the level of the patient participant, intervention team, and neurologist. Patient- and provider-level acceptability, feasibility, and appropriateness are collected via very brief validated quantitative measures [53]. Patient participants in the intervention group complete these measures before receiving the intervention and at the 3-month outcome assessment.

**Table 2:** Implementation (aim 2) end points and levels of measurement.

Concepts and measures		Levels
**Fidelity (primary: intervention adherence)**		
	Proportion meeting minimum adherence metric of 50% call participation at 12 weeks: primary implementation measure	Patient
	Percentage attendance: secondary implementation measure	Patient
	Process measures	Neurologist, patient, and intervention team
**Feasibility^a^**		
	Feasibility of Intervention Measure	Patient and neurologist
**Acceptability^a^**		
	Acceptability of Intervention Measure	Patient and neurologist
**Appropriateness^a^**		
	Intervention Appropriateness Measure	Patient and neurologist

^a^Four-item self-report scales that produce a continuous score ranging from 4 to 20, with higher scores indicating higher feasibility, acceptability, and appropriateness for the respective instrument [53].

Neurology providers at the study sites are offered a brief (<30 min) collaborative care training session in person or virtually, delivered by the PI along with members of the local intervention team. They are then offered the opportunity to participate in the implementation measure survey [53] after providing consent through a brief information sheet. Initial neurology provider training sessions were held before the study launch at the initial study sites. Additional training sessions and survey participation are offered on a rolling basis to new neurology provider staff and staff at newly participating neurology clinics within the study site networks. Neurology providers will have the opportunity to complete follow-up acceptability, feasibility, and appropriateness surveys at the study midpoint. Neurology care provider–level process measures will be collected, including whether psychotropic medication prescribing recommendations conveyed by the intervention team were carried out by the neurology providers.

### Study Intervention

#### Overview

**Figure 2:**
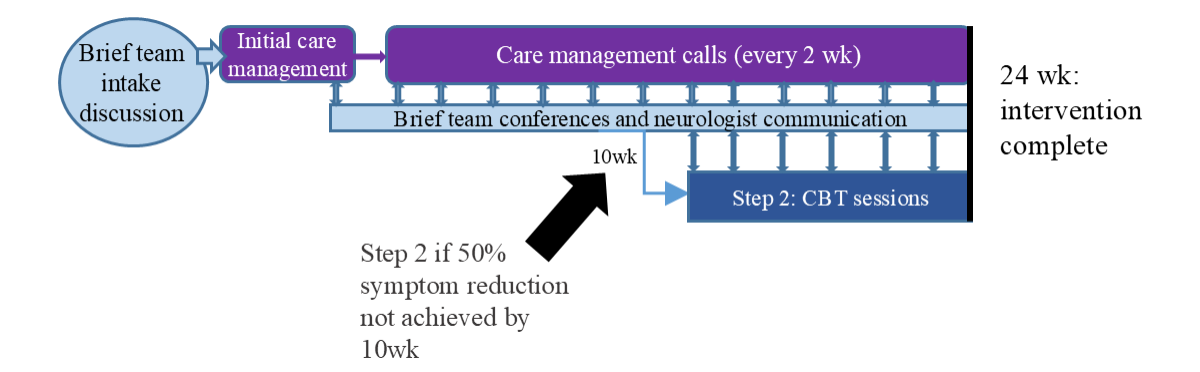
Intervention schema. CBT: cognitive behavioral therapy.

The intervention is characterized by a neurology-oriented, remotely delivered collaborative care team model for anxiety and depression management lasting 24 weeks (Figure 2). The team is composed of a care manager (either a nurse or a licensed clinical social worker) who monitors patient symptoms, seizures, and medications and provides care management support and brief therapy interventions via video or telephone calls every 2 weeks; a psychiatrist who primarily provides recommendations at virtual team meetings occurring between care management calls; and a therapist (psychologist or licensed clinical social worker) who delivers an 8-session cognitive behavioral therapy (CBT) program in step 2 of the intervention, if needed. This team communicates with the patient’s primary neurologist or neurology provider and provides recommendations for mental health medication interventions to the neurologist. This intervention is grounded in primary care and nonneurology subspecialty collaborative care models demonstrated to be more effective than usual care [20] and adapted from existing primary care and general programs at the study sites, with study intervention team members having expertise in these existing programs (family medicine–based collaborative care at the Wake Forest University School of Medicine and the VA’s Whole Health and Primary Care Mental Health Integration programs).

#### Care Management Calls and Conferences

A preintervention care conference meeting is held before each participant’s first intervention visit with the care manager, typically 1 to 2 weeks after randomization. These meetings are initial brief virtual team intake discussions that review the following information: key aspects of the enrollment MINI [47] results, baseline PHQ-9 and GAD-7 scores, and current and past psychiatric treatment history from the enrollment interview (the collection of this information at baseline serves the care team in the process of understanding what formal information could help in diagnosis and management). In addition, preliminary treatment recommendations are formulated by the collaborative care team (including the use of selected brief therapy interventions), and care management begins at the first intervention appointment that occurs a few days to a week after the initial care conference. The information provided for the initial virtual team case discussion is meant to help frame information that could be gathered by self-report as an initial step in the intervention in clinical practice or by the care manager at an initial care management visit. This provides an opportunity for the experienced mental health clinicians leading the intervention team (psychiatrists and psychologist) to prepare non–mental health care manager clinicians (nurses with coaching experience at the VA site) to deliver the intervention and collect subsequent appropriate mental health assessment information.

The initial care management session is conducted by video, in which the care manager verifies the current symptoms and past and current treatment history with the patient, reviews medication management for both anxiety and depression as well as epilepsy, establishes patient-centered treatment goals, and initiates the treatment recommendations of the collaborative care team. Medication education, when applicable and appropriate, and reinforcement of medication adherence are delivered.

Care management continues with telephone calls or video visits every 2 weeks for 24 weeks in which anxiety and depression scale scores are collected or reviewed (GAD-7 and PHQ-9 instruments for anxiety or depressive symptom severity, respectively, available electronically or on paper for completion before scheduled calls or visits or administered by interview during the call); and response to treatment, any side effects, and barriers to treatment are discussed. The mode of these visits or calls (video or telephone) is determined based on participant preference. The PHQ-9 and GAD-7 instruments were chosen because of their common use in collaborative care models and their ability to characterize symptom severity for depression and anxiety, as well as because they are NINDS epilepsy common data elements. At each intervention call, brief therapy interventions are also delivered to enhance behavioral health. Seizure frequency, side effects of antiseizure medications, and barriers to seizure medication adherence are also assessed during care management calls or visits. To enhance adherence to the intervention calls, telephone or electronic reminders occur the day before each scheduled call.

Team care includes care management conferences between care management calls or visits. These are brief meetings in which the care manager discusses updates on intervention participants since their prior care management call with the intervention team psychiatrist (and psychologist when applicable), and updated recommendations are formulated for the content of care management calls (especially the brief therapy interventions), antidepressant or psychotropic medication management recommendations to be relayed to the neurologist, and potential referrals or direct psychiatry care recommendations. The care manager facilitates communication between the collaborative care team and the neurologist and assists as needed with referrals and medication access. In addition to relaying antidepressant prescribing recommendations to the neurologist, the care manager relays information on seizure status or antiseizure medication side effects to the neurologist or neurology provider if patients are not free from seizures and side effects. Communication with the intervention care manager or psychiatrist can also be initiated by the neurologist at any time.

#### Brief Therapy Interventions for Care Management Calls

Brief therapy interventions, selected during the intervention team care management conferences, include wellness-based interventions, psychoeducation, problem-solving therapy, behavioral activation, relaxation, mindfulness, or mindful movement. A combination of these is applied, tailored to each participant. Wellness-based interventions focus on the holistic well-being of the participant, identifying areas where they feel they are doing well and where they may need some assistance [54]. Psychoeducation involves informing patients about their symptoms and diagnosis and providing relevant information on these topics throughout collaborative care [55]. Problem-solving therapy helps define areas of concern in the patient’s life and supports brainstorming potential solutions to their problems [56]. Behavioral activation involves incorporating positive activities or experiences into daily life, aiming to help participants identify positive activities they could engage in between calls to increase activity levels and decrease anxiety or depressive symptoms [57]. Relaxation techniques teach participants skills related to deep breathing, imagery, and muscle relaxation to assist with grounding and calming, providing temporary relief from tension, stress, or mental or physical pain [58]. Mindfulness encourages participants to live in the present moment by observing current thoughts and feelings, rather than dwelling on the past or worrying about the future [59]. Mindful movement involves checking in with the body and moving in a way that can help lower stress, release stagnant energy, and strengthen the mind-body connection [60]. To assist with the introduction and practice of these topics, participants are provided with related handouts from Therapist Aid (a fair-use free web-based resource for therapists, counselors, clinicians, and care managers) and freely available documents from the VA Whole Health program that are referenced during calls.

#### Step 2 CBT Intervention

If symptoms for which the participant met criterial for enrollment fail to decrease to 50% of the initial GAD-7 and PHQ-9 measures by week 10, participants move to step 2, which involves 8 sessions of brief, remote CBT by the team psychologist or clinical social worker. At the 10-week collaborative care conference, whether step 2 of the intervention is indicated will be evaluated by examining baseline and the most recent scores for the symptom category or categories for which the participant met study inclusion criteria at enrollment (anxiety only, depression only, or both anxiety and depression). If the most recent scores are not ≤50% compared to the baseline and enrollment scores, the CBT step will typically be added to ongoing care management calls. This intervention is used as a more-intensive level of care for those not showing a significant reduction in symptoms since beginning the care management calls. Each 30-minute CBT session is delivered by a licensed psychologist or licensed clinical social worker and includes the following component activities: a review of symptom screening tools (typically the GAD-7 and PHQ-9 from the most recent care management call), teaching of the main CBT topic area for the session, teaching of a technique to use at home as a tool to manage symptoms, and discussion of homework assignments for the period after the session. The first session also includes an initial content element: goal setting, identifying the targeted symptoms, and desired change if the intervention is successful. Sessions 2 to 8 include a review of homework practice from the prior session early in the appointment schedule. The main topics covered across the 8 sessions are an introduction to the CBT model (how thoughts, feelings, and behaviors interact), the use of thought records (tracking daily thoughts and feelings and reframing), the cycle of anxiety or depression through a CBT lens, the role of automatic negative thoughts or automatic thoughts, behavioral intervention through a CBT lens (behavioral activation and exposure and response inhibition), core beliefs and how they impact our thoughts and feelings, situations or stressors (antecedents) applied to thought record use, and creating a plan for the independent use of CBT concepts and tools after the conclusion of the study [61].

#### Interventionist Training and Fidelity Assessment

To minimize bias in the results from using distinct care managers and therapists for CBT delivery at the 2 sites, checklists and a manual with scripts were provided to intervention team members at both sites, and members at both sites underwent a uniform training process, including manual self-study, live training sessions delivered by intervention team lead investigators, and rating of competencies using the checklists. Care managers also completed the American Psychiatric Association web-based module “Applying the Integrated Care Approach: Skills for the Behavioral Health Care Manager” before intervention delivery. A demonstration of adequate competency on all checklist items is required before study intervention delivery by a team member. During intervention delivery, care management calls and CBT sessions are recorded using secure methods acceptable per local study site policies, and some sessions are randomly selected for review to evaluate adherence to the key components on the checklists. Further training will be provided if needed to team members to maximize the consistency of intervention delivery.

### Comparator

The control group receives usual neurology care, meaning ongoing neurology provider–recommended clinic visits, prescriptions, testing, and referrals. Mental health referrals or prescribing of antidepressants may potentially occur in this group; these types of interventions will be tracked at outcome assessments.

### Safety

To ensure study safety, individuals with active suicidal ideation, past suicide attempt, or unstable substance misuse are excluded. If there is a need for immediate treatment (eg, active suicidal ideation or active psychotic symptoms) at any point in time, staff will notify the PI or designated investigator and follow established study safety procedures [62,63] to evaluate and respond. All study team members having participant contact receive specific live training on site-specific safety procedures, along with IRB-approved safety procedure instruction documents. If needed, participants may be referred for psychiatric care, including emergent psychiatric care. Study psychiatrists and the psychologist or social worker at each site are available for input and guidance as needed. As an additional safety precaution, we will ask each participant upon study entry to identify and provide emergency contacts and their contact information. All participants will receive information about safety precautions and procedures to follow in the event that a participant becomes imminently suicidal. They will also be given telephone numbers for site-specific urgent psychiatric care and a crisis hotline. As an additional safety precaution, if any participant indicates a significant worsening in anxiety or depression scores (an increase of >1 SD on the Beck Depression Inventory–Second Edition or Beck Anxiety Inventory from baseline, 12 and 10 points, respectively [45,46]), the computer software system will facilitate identifying the worsening, and an email will be sent to the site coordinator, PI, and site psychiatrist or psychologist. Staff will be instructed to follow the study-specific safety procedures in follow-up and review with the investigator any need for additional action, such as referral for psychiatric care or to other physicians as appropriate. Safety monitoring will be conducted by the Wake Forest University School of Medicine Institutional Data Safety Monitoring Board.

### Sample Size and Power

Given the emphasis on effectiveness in our trial, we estimated the total sample size using the primary effectiveness outcome, that is, change in emotional quality of life from baseline to 6 months. With a total sample size of 60, we will have 80% statistical power to detect a moderate clinically important difference in mean change equal to 20 units [26] in the collaborative care group, compared to usual care, assuming a dropout rate as high as 13% at 6 months in the intervention group and a common SD of 24 units [24] (2-sample 2-tailed *t* test, α=.05). For our secondary outcome overall (primary implementation outcome), we will have 80% statistical power to detect a true proportion of participants of 0.8 who attend a majority of the collaborative care management calls in the first 12 weeks, assuming a sample size of 30 in the intervention group (exact binomial test, α=.10, one-sided testing vs the null proportion of 0.6) [64].

### Statistical Analysis Plan

To evaluate the primary effectiveness outcome, we will test the hypothesis that there is a clinically significant change in emotional quality of life (QOLIE-31 emotional quality of life subscale) at 6 months with the collaborative care intervention compared to usual care. The primary analysis will compare changes in subscale scores from baseline to 6 months between the intervention and control arms using multiple linear regression modeling, adjusting for study site and using an intent-to-treat approach. If the data are incomplete due to withdrawal of consent or dropout, missed visits, or missed individual data items, missing data methods will be used to compare results; specifically, a sensitivity analysis and maximum likelihood repeated measures analysis will be conducted under the missing-at-random assumption. Secondary effectiveness outcomes (anxiety, depression, and epilepsy-specific overall quality of life) will be evaluated using similar methods.

In exploratory effectiveness analyses, we plan to extend this modeling to evaluate the moderators and mediators of the collaborative care intervention effects. Potential moderators will include baseline medication adherence, cognition, and social factors such as employment and marital status. Mediators will include factors related to patient seizures (seizure frequency and severity) and treatment (medication adherence and side effects). We believe analysis of these factors to be a logical next step in follow-up of the primary and secondary results, with good potential for identifying important mechanisms to help us improve our implementation strategies and increase the effectiveness or efficiency of future interventions.

To evaluate the primary implementation outcome, we will test the hypothesis that the fidelity of the intervention, as measured by the proportion of participants in the intervention group who attend a majority of the collaborative care management calls in the first 12 weeks, is >0.6 using exact binomial testing (type I error rate ≤0.10). The corresponding CI (90% level) will be calculated based on the Wilson score method [65]. The data analysis for this project will be generated using R and SAS or STAT software (SAS Institute Inc) [36].

## Results

Enrollment for the study commenced in 2023. Due to initial slow recruitment, the inclusion and exclusion criteria were adjusted after the first 6 participants enrolled, as mentioned previously. The civilian site is initiating plans to expand enrollment beyond the Atrium Health Wake Forest Baptist region of the primary study’s health system to include neurology and epilepsy care sites throughout the Atrium Health enterprise (after EHR harmonization efforts across the 2 enterprise regions). Enrollment is expected to continue at least into 2025.

## Discussion

### Summary

This hybrid type 1 effectiveness implementation trial will evaluate a 24-week collaborative care intervention adapted from existing civilian and VA programs among adults with anxiety or depressive symptoms and PTE. The analysis of the primary outcome will demonstrate whether the collaborative care intervention results in a moderate improvement in emotional quality of life compared to usual care and thus merits immediate clinical implementation, or whether it instead merits further investigation, such as a larger trial powered to detect a minimal clinically significant difference. The implementation results will help demonstrate whether additional refinement of the intervention or implementation strategy is needed.

### Comparison to Prior Work

This study builds on a vast body of data supporting collaborative care to improve anxiety, depression, and emotional quality of life in medical samples [20] by testing it in a neurology clinic setting among patients with epilepsy. In contrast to the Program to Encourage Active, Rewarding Lives intervention in epilepsy, which involved master’s-level therapists delivering eight 50-minute in-home sessions of problem-solving therapy emphasizing physical and social activity, our study intervention targets anxiety and depression rather than depression alone and includes additional brief therapy options tailored to individual goals [24]. Collaborative care is directly aligned with existing billing codes, in contrast to various evidence-based epilepsy self-management programs for which funding uncertainty was identified as a key barrier to implementation and sustainment [66]. Our collaborative care intervention also builds on the study team’s prior work demonstrating that while many patients with epilepsy may prefer to receive mental health care in a neurology setting [18], many individuals in this setting have inadequate symptom relief despite existing antidepressant prescribing [17], arguing for neurology clinic–based interventions that include specialized mental health input, such as collaborative care. Our collaborative care intervention incorporates elements previously reported to be preferred anxiety and depression management modalities by patients in this epilepsy care setting (wellness-based interventions, antidepressants prescribed by a neurologist, therapy, and complementary or integrative techniques such as mindfulness) [18], and the collaborative care model was also well received by patients with epilepsy in a semistructured interview study [67].

### Strengths and Limitations

This is an innovative application of a hybrid effectiveness-implementation trial design and validated quantitative implementation outcome measures in the field of epilepsy. The intervention is fully remotely delivered, which may facilitate participation for patients with epilepsy, and it is grounded in an intervention that can be billed to government and commercial insurance carriers, which may facilitate next step clinical implementation if demonstrated to be effective. The seizure care–related components of the intervention are an innovative disease-specific adaptation of collaborative care for epilepsy; these components may potentially benefit overall epilepsy care.

The limitations of this study include the small, 2-site design, which may limit generalizability but which does have the advantage of including participants from 2 very different practice settings—the VA and a civilian university site—which may mitigate this limitation. The intervention teams are small, and the intervention is adapted from local programs at these clinical sites; further evaluation in a larger multisite context may be beneficial. In addition, the teams are somewhat distinct at the VA (nurses with coaching experience, psychologist, and psychiatrist) versus the civilian site (licensed clinical social worker and psychiatrist), but we attempted to mitigate this via standardized training and checklist use as well as fidelity assessment using intervention recordings and the checklist during intervention delivery. Another limitation of this trial as an implementation study is that multiple research instruments are used to collect data, which incurs a participation burden distinct from routine care practice collaborative care and may result in selection bias and deter recruitment.

### Future Directions

Depending upon the results of this trial, the next steps could include refining the intervention or implementation strategy and further testing in a larger trial or preparation for dissemination and clinical implementation. Ultimately, the future directions include using the implementation data collected in this trial to consider implementation characteristics and build toward scalability. Future testing focused on the impact of collaborative care on seizure outcomes and testing of similar interventions in mixed neurological disease samples may be warranted. Other future directions related to health policy could include examining the impact of integrated mental health care program availability on neurologist burnout and potential advocacy for increased access and resources for mental health screening and treatment. This advocacy could include specific action to advocate for more payers to cover the collaborative care billing codes, as well as other funding and resources for mental health care in specialty clinics.

### Conclusions

CoCarePTE is an innovative hybrid type 1 effectiveness-implementation trial testing a collaborative care intervention for anxiety or depression in PTE. The main results may support immediate clinical implementation of this intervention versus further refinement and testing, and the implementation results will inform implementation strategy refinement and potential planning for scalability.

## Appendix

Multimedia Appendix 1 Peer-reviewer report from Department of Defense Congressionally Directed Medical Research Programs Epilepsy Research Program.

Multimedia Appendix 2 CONSORT checklist.

## Abbreviations

CBT: cognitive behavioral therapy
CoCarePTE: Collaborative Care for Posttraumatic Epilepsy
EHR: electronic health record
FITBIR: Federal Interagency Traumatic Brain Injury Research
GAD-7: Generalized Anxiety Disorder-7
IRB: institutional review board
MINI: Mini International Neuropsychiatric Interview
NDDI-E: Neurological Disorders Depression Inventory for Epilepsy
NINDS: National Institute of Neurological Disorders and Stroke
PHQ-9: Patient Health Questionnaire-9
PI: principal investigator
PTE: posttraumatic epilepsy
QOLIE-31: Quality of Life in Epilepsy-31
REDCap: Research Electronic Data Capture
TBI: traumatic brain injury
VA: US Department of Veterans Affairs

## Data Availability

Datasets generated and analyzed during this study will be submitted to the Federal Interagency Traumatic Brain Injury Research data repository [68] and may be requested by accessing the repository.
