# Supplementary material for: Collaborative Care to Improve Quality of Life for Anxiety and Depression in Posttraumatic Epilepsy (CoCarePTE): Protocol for a Randomized Hybrid Effectiveness-Implementation Trial
Source: JMIR Res Protoc. 2024 Nov 13;13:e59329. doi: 10.2196/59329 (PMC11602765; doi:10.2196/59329)
Supplement: Multimedia Appendix 1 [file resprot_v13i1e59329_app1.pdf]

Department of Defense
U.S. Army Medical Research and Development Command
Congressionally Directed Medical Research Programs
Fiscal Year 2021 Epilepsy Research Program
Quality of Life Research Award - Funding Level 2
Peer Review Summary Statement

CDMRP Log Number: EP210036
Grants.gov ID Number: GRANT13428564
Review Panel: Quality of Life Research
Discussion Period: 09/14/2021-09/14/2021

Project Duration: 36 months
Budget Requested: $710,648
Direct Costs: $500,000
Indirect Costs: $210,648

Title: Collaborative Care to Improve Quality of Life for Anxiety and Depression in Posttraumatic Epilepsy
Principal Investigator: Heidi Munger Clary
Performing Organization: Wake Forest University Health Sciences
Contracting Organization: Wake Forest University Health Sciences

OVERVIEW

The Principal Investigator (PI) of this application proposes to assess a neurology-oriented collaborative care intervention among 60 adults with posttraumatic epilepsy and anxiety or depression who receive care at a Veterans Affairs (VA) or a university specialty clinic. The project's specific aims are (1) to assess implementation of neurology collaborative care to manage anxiety and/or depression among adults with posttraumatic epilepsy in a 2-site randomized trial, with primary implementation outcome being fidelity (patient adherence to the intervention, N = 30) and secondary outcomes including patient and neurology clinician-level validated measures of acceptability, appropriateness, and feasibility; (2) to evaluate effectiveness of collaborative care versus usual care on 6-month change in emotional quality of life (primary outcome: emotional well-being subscale of Quality of Life in Epilepsy-31 item [QOLIE-31]/short form-36 [SF-36]) along with secondary outcomes (epilepsy-specific quality of life, generic quality of life, depression and anxiety); and (3) to explore potential mediators of the effect of collaborative care, including seizure factors (seizure frequency, seizure severity) and treatment factors (adverse effects, prescription adherence).

	**Average Score**	**Standard Deviation**
**Overall Evaluation**<br>*Rating Scale: 1.0 (highest merit) to 5.0 (lowest merit)*	2.4<br>**(Good)**	0.2
**Criteria**<br>*Rating Scale: 10 (highest merit) to 1 (lowest merit)*	**Average Score**	
**Research Strategy and Feasibility**	5.0	
**Personnel**	6.3	
**Innovation and Impact**	7.0	
**Focus Area**	8.5	
**Data and Research Resource Sharing Plan**	8.0	

Procurement Sensitive Document
Do not copy or distribute without CDMRP written permission.
Prepared by SRA International, Inc., A GDIT Company, a support contractor to CDMRP

SCORED CRITERIA

Research Strategy and Feasibility
Average Score: 5.0

Scientist Reviewer A

Strengths: The applicants do a good job of laying the groundwork for their study in the Background section. They justify the importance of the target problems (anxiety/depression) in epilepsy, document the insufficient treatment of these problems in the target population, and provide a thorough discussion of the advantages of collaborative care in other patient populations. Another strength is the provision of preliminary data regarding patient preferences for treatment and barriers to comprehensive treatment perceived by physicians. There is a detailed work plan and timeline, including a 6-month start-up period, with milestones that appear feasible and appropriate. There is extensive information on the numbers of patients estimated to be eligible for the study, including a large registry of patients who have agreed to research contact, lending confidence that the proposed sample will be achieved. Alternative strategies for recruitment, in the event that the primary sources do not pan out, are also discussed.

The plan to deliver the intervention remotely is a major strength, as this modality is preferred by many patients and overcomes significant barriers to mental health care. The relative ease of remote administration should also facilitate further study and implementation of the model. As another strength, the outcome measures selected to assess the effects of the intervention on mental health and quality of life (Aim 2) are well validated and appropriate to the aims and hypotheses addressing these constructs. Of the 3 aims, this second one is the strongest, as it will directly examine the effects of 24 weeks of collaborative care on health-related quality of life and emotional function.

Weaknesses: Perhaps the most significant weaknesses in the research strategy pertain to the intervention(s). The details of the care management calls are vaguely described; “wellness” and “education” are mentioned in passing, but there is no overarching conceptual model or even an outline of the content of the calls or their intended duration. Similarly, the stage 2 intervention is labeled cognitive behavioral therapy (CBT), but this is an umbrella term that could include a wide variety of specific practices. There is no justification provided for the expectation that the planned 6 to 8 30-minute remote sessions would effect significant improvement in medication-refractory depression/anxiety in this population. The “usual care” control condition is completely unspecified, and there is no plan to measure the treatments received by the control group either in a neurology clinic or outside of it, although patients assigned to control conditions in a clinical trial may be more apt to seek outside treatment. There is no plan to collect process variables, such as problems/solutions discussed in calls, length of calls, or patients’ adoption of wellness suggestions or various CBT strategies, although such data could inform further refinements of the treatment. Another treatment-related weakness is that while the application uses the term “fidelity” to refer to patient adherence, there is no plan to measure the more usual type of fidelity, ie, clinician adherence to the treatment manual or model. In a related point, the investigators have missed an excellent opportunity to improve fidelity by using one clinician to treat patients at both sites, which is readily done with remote treatments. Having 2 different clinicians administering the care management, and yet a third clinician at the civilian site delivering the CBT, increases the probability of “therapist effects” confounding the analysis. Taken together, these weaknesses in specification, delivery, and measurement of the interventions are quite likely to add noise and/or bias to the interpretation of treatment effects.

Other weaknesses include the absence of traumatic brain injury (TBI)-related variables measured with well-validated instruments. Although the common data elements include a well-researched screening instrument to establish the occurrence of TBI by self-report, The Ohio State University (OSU) TBI

Procurement Sensitive Document
Do not copy or distribute without CDMRP written permission.

identification (ID) interview, the applicants are using a screening tool developed by the VA site PI and tested in one study on a relatively small sample. There is no plan to categorize TBI by type (open/closed), etiology, or severity, using standard indices based on depth/duration of loss/alteration of consciousness, and no plan to verify TBI data using medical records. These are standard practices in TBI research. The baseline assessment includes no measure of cognitive function, despite the fact that memory and executive function have been shown to be important moderators of treatment response in TBI trials. (As an example of how this could be accomplished in the present study, the Brief Test of Adult Cognition by Telephone [BTACT] telephone battery can be delivered in 10 to 12 minutes, measures both of these functions, and is a designated common data element for TBI.)

With regard to the aims and hypotheses, Aim 2, focusing on treatment effectiveness at 24 weeks, may be partially dependent on the outcome of Aim 1, which is concerned with patient attendance at the care management calls for the first 12 weeks. That is, if the hypothesis for Aim 1 is not met—fewer than 60% of participants attend the majority of calls—then treatment effectiveness might be expected to suffer. It is unclear what steps, if any, will be taken by the treatment team to promote attendance at the calls, which could be affected by a number of factors unrelated to a patient's clinical status or motivation to attend (scheduling issues, problems with phone or video access, etc). The focus of Aim 3 appears to be on the impact of medical issues only (seizure- and medication-related factors), whereas in the moderate-severe TBI population, neuropsychological and psychosocial factors would be equally if not more important to include in an exploration of moderator/mediator effects.

The statistical analysis is unclear with regard to a few points. First, it is not stated how many neurologists will contribute data on the implementation measures gathered in Aim 1, which would be relevant to evaluating both power and generalizability. Second, there are 2 primary outcome measures for Aim 2, but the expected effect size and power analysis appear to be based on only one, the SF-36.

Regarding the sample composition, participants are to be excluded for "active ongoing treatment with a psychiatrist," but it might also be important to exclude for current use of antidepressants prescribed by any type of physician. Also, it is not stated how "inadequate cognition to complete self-report instruments" (an exclusion criterion) will be determined.

Scientist Reviewer B

Strengths: The scientific rationale for this project is well supported by prior examples of the implementation of collaborative care in civilian and VA settings. This project identifies a significant gap in care and provides good justification for how the project aims could lead to filling the gap in care for people with posttraumatic epilepsy (PTE) and mental health issues. The hypotheses are well developed and address QOL for civilians, military, and veterans with PTE through improving the current care pathways for anxiety and depression.

The plan for recruitment is appropriate, and there is precedent for success in recruiting subjects with anxiety and depression. The inclusion and exclusion criteria are clearly identified and appropriate; they do not exclude individuals with a concomitant diagnosis of PNES and plan to analyze as a covariable. The method for randomization is addressed.

The intervention and timeline for delivery of the intervention are laid out in logical stepwise fashion with good integration of team members throughout and plans for shared communication and integration of telehealth (video and telephone encounters).

An outline for statistical analysis is provided with statistical power considerations included. The milestones set by the investigators are reasonable and achievable. There is a plan in place for telephone

Procurement Sensitive Document
Do not copy or distribute without CDMRP written permission.

outcome collection if electronic questionnaires are not returned. The participant compensation is addressed and appropriate. Safety plan consideration is included in the methods.

Weaknesses: This study does not involve caregivers or address QOL measures in caregivers; intervention and measures are restricted to PTE subjects alone. No specifics are provided around CBT interventions to be implemented, and a variable delivery of 6 to 8 sessions is included as opposed to a consistent number of sessions. The “brief” or “very brief” descriptors of interactions are included in the proposal’s outline of the intervention, but it is not clear exact time commitment these descriptors are referencing. It is also unclear whether checklists or scripts will be provided to interventionists (care managers). Experience of the clinical social worker in working with patients with a history of TBI or PTE is not provided, and the application does not appear to describe any training to remedy a deficit of experience.

The recruitment process of neurologists at each center, who will participate, is not clearly outlined. Pharmacy records may not accurately reflect medication adherence as other barriers to adherence in patients with PTE and mental health disorders are known to exist. The neurologist is communicated to by the team but is not a part of team decision-making meetings; this could be limiting to recommendations being made if there is not some real-time, of-the-moment feedback regarding participants’ current seizure control, antiseizure medicine tolerance/adherence, etc.

The initial care management session is described as being via video, but no timeline for the session or structure or materials to be reviewed are provided in this proposal. No tools (calendars, automated reminders) are described as being provided to the participants to help them with adherence to the intervention over several weeks.

It is unclear how seizure frequency will be documented by participants and reported to a care manager. For example, it is unclear if participants will be using a paper or digital seizure diary. Similarly, it is unclear how participants will track any medication side effects. A 10% attrition may be an underestimate for an intervention of this length and with a cohort impacted by mental health disorders and, for some subjects, likely cognitive dysfunction as well.

Scientist Reviewer C

Strengths: The PI is investigating an important issue of underrecognition and undertreatment of depression and anxiety and their impact on quality of life in posttraumatic epilepsy patients. The PI is directly addressing barriers to specialty mental health care access and physician time-related barriers. The scientific rationale is clearly stated, including the prevalence of depression and anxiety in this patient population, barriers to accessing mental health, and the potential advantages of collaborative care.

The preliminary data are meticulously presented and show that usual neurology care does not improve quality of life and support the need of the alternative approach of collaborative care proposed by the investigators. Preliminary data show that the investigators have implemented neurology clinic-oriented interventions to address anxiety and depression in epilepsy and support the feasibility of the project.

The hypotheses are clearly stated and developed based on the preliminary data. The specific aims are well defined and independent of each other. The study design is well thought out and described, including relevant inclusion and exclusion criteria. Methods of enrollment and screening are thorough. Standardized measures for epilepsy, depression, and anxiety as well as outcomes are utilized. The analysis plans and power calculation are sound.

The investigators appropriately acknowledge potential for insufficient recruitment and have a risk mitigation strategy of expanding to general neurology and other affiliated clinics. The inability of the

Procurement Sensitive Document
Do not copy or distribute without CDMRP written permission.

sample size to detect small differences has been acknowledged, and that it would require further investigation. Milestones and study time frame appear sound.

Weaknesses: Details of the type of CBT interventions are not well defined, along with lack of specific details on the education and wellness plans. The investigators have not detailed how the severity or type of TBI will be quantified. The sample size would not be sufficient to detect small-scale differences or smaller effect sizes. Follow-up of 6 months may not be enough to demonstrate the impact of the intervention. Data to support the estimated dropout rate are not clear and may be an underestimate.

Biostatistician Reviewer

Strengths: The preliminary data support feasibility of the trial. Outcome measures and their measurement time points are clearly specified in the proposal. Sample size calculations have taken attrition rate into consideration. The analysis method for analyzing the primary outcome is appropriate.

Weaknesses: The application does not mention how to adjust for multiplicity due to 2 primary outcomes. The success of the study under 2 primary outcomes and the associated hypotheses (null and alternative) need to be described in more detail. The proposed analysis methods for the repeated-measures outcomes are not provided. It is unclear which causal relationship in the mediation analysis will be assessed. The outcome of this causal relationship needs to be specified. Although the proposal provides information on an alternative analytic method for missing data, it is unclear what "adjusted maximum likelihood repeated measures analysis" is. A more specific description or a citation needs to be provided.

Personnel
Average Score: 6.3

Scientist Reviewer A

The PI, Heidi Munger Clary, received an MD degree from Northwestern University in 2007 and a master of public health degree from the same institution in the same year. She completed a residency in neurology in 2011, a fellowship in clinical neurophysiology in 2012, and a fellowship in epilepsy in 2013, all at Columbia University in New York. Dr Munger Clary joined the faculty of Wake Forest School of Medicine in Winston-Salem, North Carolina, as an assistant professor of neurology in 2013. As of 2016, she holds the position of associate professor of neurology at Wake Forest.

Strengths: The PI has been investigating the topic of this proposal for some time, including a K-type award granted in 2019, and the majority of her approximately 15 peer-reviewed papers are concerned with epilepsy. The investigative team at Wake Forest (Dr Munger Clary, Dr Snively, and Dr Kimball) has collaborated on previous research, including the preliminary studies leading to this proposal, lending confidence that they will have a smooth working relationship. There is a biostatistician with ample time dedicated to the project (Dr Snively). Overall, the team appears to have a strong background in epilepsy and is experienced in dealing with the mental health problems to be addressed by the intervention.

Weaknesses: The assembled team is not particularly strong with regard to TBI. Dr Hurley is identified as the team's TBI expert, but although she is an accomplished and senior neuropsychiatrist, her work is quite eclectic within that field, and she does not appear to have a particular concentration on TBI. None of the PI's published work on epilepsy deals with posttraumatic epilepsy, and Dr Hurley is second author on only 1 paper on that topic, published in 2014. No one connected to the proposal has identified prior grant funding or clinical expertise with moderate-severe TBI, which accounts for the preponderance of posttraumatic epilepsy. Another potential weakness is that some of the effort levels may be insufficient to

manage the trial, particularly Dr Munger Clary's effort (.15 full-time equivalent [FTE]) and the .05 FTE committed by each of the VA site PIs.

Scientist Reviewer B

Strengths: The PI is an associate professor of neurology and an experienced clinical researcher in the field of epilepsy. She is regarded as a leader in epilepsy quality measures and developing clinical tools to address psychosocial comorbidities (anxiety and depression) in epilepsy. She is well suited to serve as the Principal Investigator for this project. The Coinvestigators come with a breadth of training, knowledge, and experience that will aid in bringing to fruition the project goals. There is an experienced biostatistician with time allocated to complete the analyses and facilitate the upload of data into Federal Interagency TBI Research (FITBIR).

Weaknesses: The social worker who will play a significant role in carrying out this project has not been identified, though the proposal suggests there is a pool of social workers with experience in collaborative care to draw from at the Wake Forest Family Medicine Clinic. This individual may require additional training in the needs/care of persons with TBI and epilepsy to be most effective in the care manager and counselor roles.

Scientist Reviewer C

Strengths: The study team's background and related experience make it well qualified to perform the proposed work. Dr Munger Clary (PI) is well trained, including an MD/MPH (2007), clinical neurophysiology fellowship (2012), and the National Institute of Neurological Disorders and Stroke (NINDS) Clinical Trials Methodology Course with distinction. Dr Munger Clary has a track record of first-author publications related to the proposed project and a recently completed KL2 grant, making the proposed project a natural next step. Dr Munger Clary has high potential to lead the project and team. Robin Hurley, MD (1990) completed a residency in psychiatry (1994) and is a renowned researcher in neuropsychiatry, serving as PI for several multisite studies. Richard Kennerly has a PhD (2006) and fellowship (2008) in clinical psychology biofeedback behavioral medicine and experience in providing telehealth psychological and neuropsychological evaluations. Dr Kennerly has experience at the VA in collaborative care and hence is well positioned to provide expertise in the collaborative care implementation. James Kimball, MD (1998) has training in psychiatry (2002) and psychosomatic medicine (2003). Dr Kimball has extensive research experience in randomized trials in depression and will bring in both research and clinical expertise along with supervision of the social worker and care coordinator. Dr Beverly Snively has a PhD in biostatistics (1997) and has experience in neurology and psychiatry-related clinical trials and longitudinal observational analyses. The composition of the study team is appropriate and balanced, with experts in epilepsy, traumatic brain injury, and research in neuropsychiatry.

Weaknesses: The PI's level of effort may be low for the proposed work in the proposed timeline. The study team does not include an investigator with TBI and specifically PTE-related research experience.

Innovation and Impact
Average Score: 7.0

Scientist Reviewer A

Strengths: The application is innovative and potentially impactful in 2 ways. First, the applicants propose to implement a collaborative care model in the context of routine neurology treatment for PTE. As argued in the proposal, this model has shown effectiveness in numerous populations, but its use in this target

population represents an innovation. If successful, the treatment model stands to have a significant impact on emotional function and quality of life for those with PTE. Second, the delivery of all interventions via remote means is a significant innovation, which stands to have a positive impact on civilians and service members who experience barriers to mental health services related to geographic limitations or perceived stigma.

Weaknesses: The impact of this treatment model, even if the trial is successful, will be mitigated by the assumption that patients with PTE and anxiety/depression will prefer to start treatment with medication, followed by brief CBT if symptoms do not improve. The investigators' own preliminary data show quite a variety of treatment preferences, which are not fully incorporated into this somewhat rigid treatment model. There have been studies of patient needs and preferences for mental health modalities in the TBI population, but these have not been cited. In addition, uptake into clinical practice could be affected by the additional costs of treatment, but cost/benefit analysis is not mentioned as a potential next step.

Scientist Reviewer B

Strengths: This proposal includes elements of innovation. The investigators incorporate translational science techniques designed to speed the transition of evidence-based therapies into real-world care settings. The study uses principles of implementation science novel to epilepsy research. Given the success of the collaborative care model in other clinical environments and the potential for reimbursement via insurance, this model has good potential for scalability and sustainability if found to be beneficial in the PTE population The proposal addresses a well-known care gap for the treatment of anxiety and depression in the PTE population. By proposing a neurology clinic-oriented solution that meets patient preferences for receiving care in the neurology clinic but incorporates specialty mental health input efficiently, the investigators suggest this has potential to decrease behavioral health provider shortages. There is potential for positive impact in addressing mental health and QOL in military and civilian communities if the collaborative care model is found beneficial to improving anxiety, depression, and QOL in military, veteran, and civilian PTE populations.

Weaknesses: This proposal does not include feedback from caregivers or measures of burden or QOL in caregivers who have a loved one with PTE participating in the research study. Examining a collaborative care model that involves the caregiver would bring additional innovation and potentially greater impact.

Scientist Reviewer C

Strengths: The research proposal is creative in its design, going beyond efficacy studies and basic-to-clinical translation science and using implementation science. The investigators are challenging the usual neurology care for patients with posttraumatic epilepsy and depression and proposing an innovative collaborative care intervention. The investigators are evaluating the impact of their intervention in a real-world setting, also making this a high-impact study. The research innovation can have a significant beneficial impact on the military, veteran, and civilian communities. The proposed intervention may overcome mental health specialist shortages and other barriers to accessing mental health specialists.

Weaknesses: It is unclear what the potential costs of the CBT would be when implemented clinically.

Consumer Reviewer

Strengths: Anxiety and depression are prevalent comorbidities of those living with epilepsy, whether caused by PTE or otherwise. Neurologists, by training, focus therapeutic treatments on medications, sometimes with some emphasis on diet as well (in order to combat side effects of medications, primarily). Even in epileptology, emphasis will still be observed on medications and/or surgical interventions with

Procurement Sensitive Document
Do not copy or distribute without CDMRP written permission.

little attention paid to mental health. Collaborative care has already shown efficacy in other patient settings and, in some cases, has even shown cost efficiency. The idea that collaborative care could be integrated into neurology care for those diagnosed with PTE does, indeed, suggest promise to positively impacting quality of life of those living with PTE (military, veteran, and civilian communities alike) based on other implementations and studies found during this review. The inclusion of telehealth in the proposal is also a key strength of this proposal, knowing that those living with epilepsy may not drive or may struggle to obtain reliable transportation. This proposal strongly aligns to the key focus areas of QOL's program announcement (PA) in that it will address interventions related to behavioral health as well as potential impacts related to psychosocial and overall wellness.

Weaknesses: There does seem to be strong evidence to support the reasons to test collaborative care in the neurological setting, though as the applicants disclaim, shortages in mental health professionals may be a limiting factor to the availability of these services, as well as what the actual treatments would involve. It is also unclear in the narratives how study participants would pay for the collaborative care services post study; with the prevalence of unemployment in those living with epilepsy, that information would be helpful in evaluating this aspect of the proposal. Also noted was the absence of caregivers in the model.

Focus Area
Average Score: 8.5

Scientist Reviewer A

Strengths: The proposal is extremely well aligned with the focus area. The investigators propose to test an intervention targeting behavioral health and, to some extent, wellness; they will analyze the effects of medication side effects during the trial, and they will use quality-of-life measures to assess the primary outcome of the trial.

Weaknesses: No weaknesses were noted.

Scientist Reviewer B

Strengths: The focus area specific to the FY21 Epilepsy Research Program (ERP) is understanding and improving the quality of life of individuals with PTE, their families, and their caregivers. The proposed study is very well aligned with the category of behavioral health (anxiety and depression).

Weaknesses: A weakness is there is no integration of caregivers/families in this proposal.

Scientist Reviewer C

Strengths: The proposed study is well aligned with several domains of the FY21 ERP QOL focus area. The research is strongly aligned with behavioral health and wellness. The research also appropriately addresses the domain of reducing health disparities (by providing greater access to mental health).

Weaknesses: While the investigators list medication side effects as another focus area the research is aligned with, there is little detail on how this aspect will be approached in the research strategy.

Data and Research Resource Sharing Plan
Average Score: 8.0

Procurement Sensitive Document
Do not copy or distribute without CDMRP written permission.

Scientist Reviewer A

Strengths: The data sharing plan is thoughtful and comprehensive, with adequately detailed plans to upload data to FITBIR and clinicaltrials.gov and to share data as needed with outside investigators. The milestones for data sharing and the plan to make the data available following the period of performance are appropriate.

Weaknesses: The absence of TBI etiology/severity data will limit cross talk with other studies (which is part of the purpose of FITBIR data sharing). Also, the disseminaton plan notes only publications and presentations targeting neurology and epilepsy audiences, not those intended to reach clinicians and researchers concerned with TBI.

Scientist Reviewer B

Strengths: The data obtained from the proposed research are appropriate for FITBIR data sharing, and outcomes and variables for this proposal were selected for maximal potential alignment with the TBI common data elements. The investigators provide a plan for how the data from this study could be made publicly available. They provide a description of the milestones for accomplishing data sharing goals. The study team's biostatistician has been identified as the person responsible for data submission to FITBIR and clinicaltrials.gov.

Weaknesses: No significant weaknesses were identified.

Scientist Reviewer C

Strengths: The data and research resource sharing plan is well detailed. The investigators clearly state that they will be compliant with the DOD and clinicaltrials.gov requirements.

Findings will be made available to the FITBIR information system, and the process for doing so has been clearly laid out. The posting of data and results in FITBIR and on clinicaltrials.gov will allow access to the data past expiration of study funding.

The milestones for making the data and research resource(s) available seem appropriate. In accordance with clinicaltrials.gov requirements, the investigators will post study design information, study protocol, informed consent, and baseline and primary and secondary outcome results according to the timing requirements of clinicaltrials.gov.

A description of the type of data or research resource(s) to be made publicly available is provided. A plan for data sharing has also been outlined, including ways to ensure HIPAA compliance.

Weaknesses: None were identified.

UNSCORED CRITERIA

Budget

Scientist Reviewer A

The direct costs do not exceed the allowable amount. The budget appears appropriate overall. Items that might be a bit excessive include the salary amounts for 2 REDCap developers (1 per site) and the time allotted to the project statistician (.10 FTE for all 3 grant years, which is unusual).

Scientist Reviewer B

The requested funds fall within the allowable costs for a level 2 project. The expenditures for salaries, travel, and subject fees appear reasonable for the proposed project.

Scientist Reviewer C

The direct costs are within the allowable direct costs ($500,000). The budget is appropriate for the proposed research.

Intellectual Property

Scientist Reviewer A

Intellectual property issues are not discussed in the application.

Scientist Reviewer B

There is no intellectual property/commercialization plan included in the proposal.

Scientist Reviewer C

There is no intellectual property plan included in the proposal.

Environment

Scientist Reviewer A

The scientific environment appears appropriate (ie, excellent). Letters are included that confirm the enthusiastic support of both participating institutions.

Scientist Reviewer B

It is clear that the scientific environment and support for the proposed research are appropriate.

Scientist Reviewer C

Wake Forest University has a well-established comprehensive epilepsy center and meets the level IV guidelines of the National Association of Epilepsy Centers, with a large epilepsy patient population making the environment clinically appropriate for the proposed research. Appropriate scientific and academic resources include the Department of Biostatistics, Department of Implementation Sciences, and the Wake Forest Clinical and Translational Science Institute (CTSI).

The Salisbury VAMC is the 12th largest in the nation and will also provide a rich clinical and scientific environment. The Mental Illness Research, Education, and Clinical Center (MIRECC) has a central database of post-911 veterans with epidemiological and neuropsychological data. Under existing institutional review board (IRB) approval, this can be used for the proposed research.

The Wake Forest University and VAMC leadership are very supportive of the proposed application and research team, with strong letters of endorsement.

Procurement Sensitive Document
Do not copy or distribute without CDMRP written permission.

Application Presentation

Scientist Reviewer A

The application was clearly written and free of typographical errors. One clerical error was noted: the inadvertent inclusion of a draft budget justification page for the VA site. The correct justification document was present a few pages later, however. The presentation did not affect the review. The application adequately reflected knowledge of and respect for affected individuals, particularly those with epilepsy, with somewhat less knowledge about the impact of TBI.

Scientist Reviewer B

The application was clearly written and free of typographical errors, and the figures and tables provided were helpful (some more than others). The application reflected knowledge of the mental health needs of patients with posttraumatic epilepsy in military and civilian populations.

Scientist Reviewer C

The application is well written and organized in presentation, facilitating the review process. The application demonstrates strong knowledge and respect for the needs of affected individuals. The needs of caregivers and families are not addressed in detail.

Procurement Sensitive Document
Do not copy or distribute without CDMRP written permission.
