# Supplementary material for: Collaborative Care to Improve Quality of Life for Anxiety and Depression in Posttraumatic Epilepsy (CoCarePTE): Protocol for a Randomized Hybrid Effectiveness-Implementation Trial
Source: JMIR Res Protoc. 2024 Nov 13;13:e59329. doi: 10.2196/59329 (PMC11602765; doi:10.2196/59329)
Supplement: Multimedia Appendix 2 [file resprot_v13i1e59329_app2.pdf]

CONSORT 2010 checklist of information to include when reporting a randomised trial*
(adapted with addition of extension elements for nonpharmacologic trials highlighted gray)

Section/Topic	Item No	Checklist item	Reported on page No
**Title and abstract**			
	1a	Identification as a randomised trial in the title	1
	1b	Structured summary of trial design, methods, results, and conclusions (for specific guidance see CONSORT extension for abstracts for NPT trials)	2
**Introduction**			
Background and objectives	2a	Scientific background and explanation of rationale	3
	2b	Specific objectives or hypotheses	4
**Methods**			
Trial design	3a	Description of trial design (such as parallel, factorial) including allocation ratio. When applicable, how care providers were allocated to each trial group.	4
	3b	Important changes to methods after trial commencement (such as eligibility criteria), with reasons	6,18
Participants	4a	Eligibility criteria for participants. When applicable, eligibility criteria for centers and for care providers.	5-6
	4b	Settings and locations where the data were collected	4-5
Interventions	5	The interventions for each group with sufficient details to allow replication, including how and when they were actually administered. Precise details of both the experimental treatment and comparator.	13-16
	5a	Description of the different components of the interventions and, when applicable, description of the procedure for tailoring the interventions to individual participants.	13-16
	5b	Details of whether and how the interventions were standardized.	16
	5c	Details of whether and how adherence of care providers to the protocol was assessed or enhanced.	16
	5d	Details of whether and how adherence of participants to interventions was assessed or enhanced.	14
Outcomes	6a	Completely defined pre-specified primary and secondary outcome measures, including how and when they were assessed	8,10,12-13
	6b	Any changes to trial outcomes after the trial commenced, with reasons	n/a
Sample size	7a	How sample size was determined. When applicable, details of whether and how the clustering by care providers or centers was addressed.	17
	7b	When applicable, explanation of any interim analyses and stopping guidelines	n/a

Randomisation:			
Sequence generation	8a	Method used to generate the random allocation sequence	7
	8b	Type of randomisation; details of any restriction (such as blocking and block size)	7
Allocation concealment mechanism	9	Mechanism used to implement the random allocation sequence (such as sequentially numbered containers), describing any steps taken to conceal the sequence until interventions were assigned	7
Implementation	10	Who generated the random allocation sequence, who enrolled participants, and who assigned participants to interventions	7
Blinding	11a	If done, who was blinded after assignment to interventions (for example, participants, care providers, those assessing outcomes) and how. If done, who was blinded after assignment to interventions (e.g.,participants, care providers, those administering co-interventions, those assessing outcomes) and how.	7
	11b	If relevant, description of the similarity of interventions	n/a
	11c	If blinding was not possible, description of any attempts to limit bias	n/a
Statistical methods	12a	Statistical methods used to compare groups for primary and secondary outcomes. When applicable, details of whether and how the clustering by care providers or centers was addressed.	17-18
	12b	Methods for additional analyses, such as subgroup analyses and adjusted analyses	17-18
**Results**			
Participant flow (a diagram is strongly recommended)	13a	For each group, the numbers of participants who were randomly assigned, received intended treatment, and were analysed for the primary outcome. The number of care providers or centers performing the intervention in each group and the number of patients treated by each care provider or in each center.	n/a
	13b	For each group, losses and exclusions after randomisation, together with reasons	n/a
	13c	For each group, the delay between randomization and the initiation of the intervention. Details of the experimental treatment and comparator as they were implemented.	n/a
Recruitment	14a	Dates defining the periods of recruitment and follow-up	n/a
	14b	Why the trial ended or was stopped	n/a
Baseline data	15	A table showing baseline demographic and clinical characteristics for each group. When applicable, a description of care providers (case volume, qualification, expertise, etc.) and centers (volume) in each group.	n/a
Numbers analysed	16	For each group, number of participants (denominator) included in each analysis and whether the analysis was by original assigned groups	n/a
Outcomes and estimation	17a	For each primary and secondary outcome, results for each group, and the estimated effect size and its precision (such as 95% confidence interval)	n/a
	17b	For binary outcomes, presentation of both absolute and relative effect sizes is recommended	n/a
Ancillary analyses	18	Results of any other analyses performed, including subgroup analyses and adjusted analyses, distinguishing	n/a

		pre-specified from exploratory	
Harms	19	All important harms or unintended effects in each group (for specific guidance see CONSORT for harms)	n/a
**Discussion**			
Limitations	20	Trial limitations, addressing sources of potential bias, imprecision, and, if relevant, multiplicity of analyses. In addition, take into account the choice of the comparator, lack of or partial blinding, and unequal expertise of care providers or centers in each group.	19
Generalisability	21	Generalisability (external validity, applicability) of the trial findings. Generalizability (external validity) of the trial findings according to the intervention, comparators, patients, and care providers and centers involved in the trial	19
Interpretation	22	Interpretation consistent with results, balancing benefits and harms, and considering other relevant evidence	n/a
**Other information**			
Registration	23	Registration number and name of trial registry	2,5
Protocol	24	Where the full trial protocol can be accessed, if available	n/a
Funding	25	Sources of funding and other support (such as supply of drugs), role of funders	5

Citation: Schulz KF, Altman DG, Moher D, for the CONSORT Group. CONSORT 2010 Statement: updated guidelines for reporting parallel group randomised trials. BMC Medicine. 2010;8:18.
© 2010 Schulz et al. This is an Open Access article distributed under the terms of the Creative Commons Attribution License (http://creativecommons.org/licenses/by/2.0), which permits unrestricted use, distribution, and reproduction in any medium, provided the original work is properly cited.

*We strongly recommend reading this statement in conjunction with the CONSORT 2010 Explanation and Elaboration for important clarifications on all the items. If relevant, we also recommend reading CONSORT extensions for cluster randomised trials, non-inferiority and equivalence trials, non-pharmacological treatments, herbal interventions, and pragmatic trials. Additional extensions are forthcoming: for those and for up-to-date references relevant to this checklist, see www.consort-statement.org.
